# Supplementary figures and images for: Determinants of return to HIV treatment after interruption on ART among HIV positive clients in Katakwi District, Uganda
Source: PLoS One. 2026 Feb 4;21(2):e0337637. doi: 10.1371/journal.pone.0337637 (PMC12872005; doi:10.1371/journal.pone.0337637)

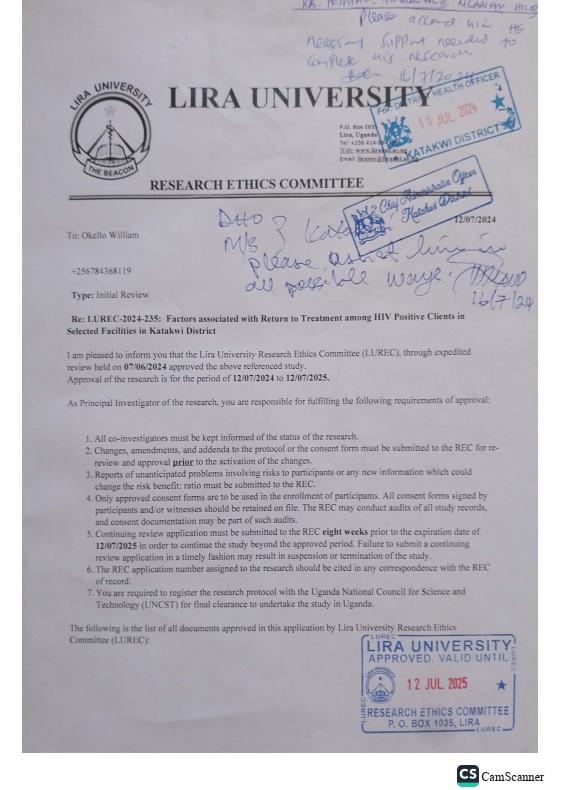

Supplement: S1 File — (DOCX) [file pone.0337637.s002.docx]
