# Supplementary material for: Determinants of return to HIV treatment after interruption on ART among HIV positive clients in Katakwi District, Uganda
Source: PLoS One. 2026 Feb 4;21(2):e0337637. doi: 10.1371/journal.pone.0337637 (PMC12872005; doi:10.1371/journal.pone.0337637)
Supplement: Attachment — Submitted filename: Response to the Reviewer {20 May 2025}.docx [file pone.0337637.s004.docx]

**Subject; Response to reviewer’s comments**

**Dear Editor.**

We appreciate the guidance and we have shown in this response letter how this has been handled. We looked forward to your favourable editorial decision.

We've checked your submission and before we can proceed, we need you to address the following issues:

1. Please include a separate caption for each figure in your manuscript.

**Response; This has been done in the manuscript see line 167 page 7**
